# Vascular Smooth Muscle Cells: A Therapeutic Target in Atherosclerosis

**DOI:** 10.31083/RCM28240

**Published:** 2025-06-26

**Authors:** Lingna Zhao, Li Zhao, Deshen Liu, Fangze Huang, Qinbao Peng, Jun Lu, Jiaguo Zhou, Shaoyi Zheng, Xiu Liu

**Affiliations:** ^1^Department of Cardiovascular Surgery, Nanfang Hospital, Southern Medical University, 510515 Guangzhou, Guangdong, China; ^2^Department of Pharmacology, Cardiac and Cerebral Vascular Research Center, Zhongshan School of Medicine, Sun Yat-sen University, 510080 Guangzhou, Guangdong, China

**Keywords:** atherosclerosis, vascular smooth muscle cell, vascular smooth muscle cell-derived foam cell, phenotypic switching, apoptosis, autophagy

## Abstract

**Background:**

Vascular smooth muscle cells (VSMCs) are involved in atherosclerotic plaque development. The formation of VSMC-originated foam cells, phenotypic switching, and VSMC proliferation, migration, apoptosis, and autophagy play different roles in atherosclerosis (AS).

**Main Body:**

Foam cell formation promotes the generation and evolution of atherosclerotic plaques. The VSMC phenotype, switching from contractile to other forms, is important in the formation and progression of AS. VSMC proliferation, migration, and apoptosis affect the stability of atherosclerotic plaques through the fibrous cap. VSMC proliferation and migration can increase the thickness of the fibrous cap of the plaques, which protects plaques from rupture and is beneficial for slowing the occurrence of advanced lesions. However, apoptosis can accelerate plaque rupturing and trigger severe cardiovascular disease. The autophagy of VSMCs has a protective influence on safeguarding cellular homeostasis in the early stages of AS. However, increased autophagy of VSMCs in the late stages of AS can lead to cell death, thereby affecting the stability of late-stage plaques. This review comprehensively reviews recent research on genetic proteins and mechanisms influencing various aspects of VSMCs, including VSMC-derived foam cells, phenotypic switching, proliferation, migration, apoptosis, and autophagy. Additionally, this review aimed to examine the implications of VSMCs for AS and discussed several regulators that can impact the progression of this condition. Our review thoroughly summarizes the latest research developments in this field.

**Conclusion:**

Based on the vital role of VSMCs in AS, this review provides an overview of the latest factors and mechanisms based on VSMC-derived foam cells, phenotype switching, proliferation, migration, apoptosis, and autophagy. The review also introduces certain regulators that can inhibit the development of AS. An understanding of the role of VSMCs aids in identifying new targets and directions for advancing innovative anti-atherosclerotic therapeutic regimens and provides new insights into the development of treatments for AS.

## 1. Background

Atherosclerosis (AS), a chronic inflammatory disease, is characterized by an overabundance of lipid deposition in the intima of the main arteries, which is detrimental to the heart and blood vessels [1]. Cardiovascular diseases caused by AS significantly endanger human life and health. The formation of AS is complex, involving numerous cellular mechanisms and pathways, and is characterized by lipid accumulation, fibrous cap formation, and necrotic nuclei [2]. Vascular smooth muscle cells (VSMCs), endothelial cells, and macrophages are closely associated with AS lesions [3]. Extensive research on AS has revealed that VSMCs are crucial to the formation and development of AS. More than half of foam cells in AS plaques originate from VSMCs rather than macrophages [3]. In addition, the phenotypic switching, proliferation and migration, apoptosis, and autophagy of VSMCs contribute to the occurrence and development of AS via different mechanisms [4]. Here, we focus on the role and various mechanisms of VSMC-derived foam cells, phenotypic switching, proliferation and migration, apoptosis, and autophagy of VSMCs in the development of AS.

## 2. VSMC-derived Foam Cells

### 2.1 VSMC-derived Foam Cell Formation in the Development of AS 

**Fig. 1.**
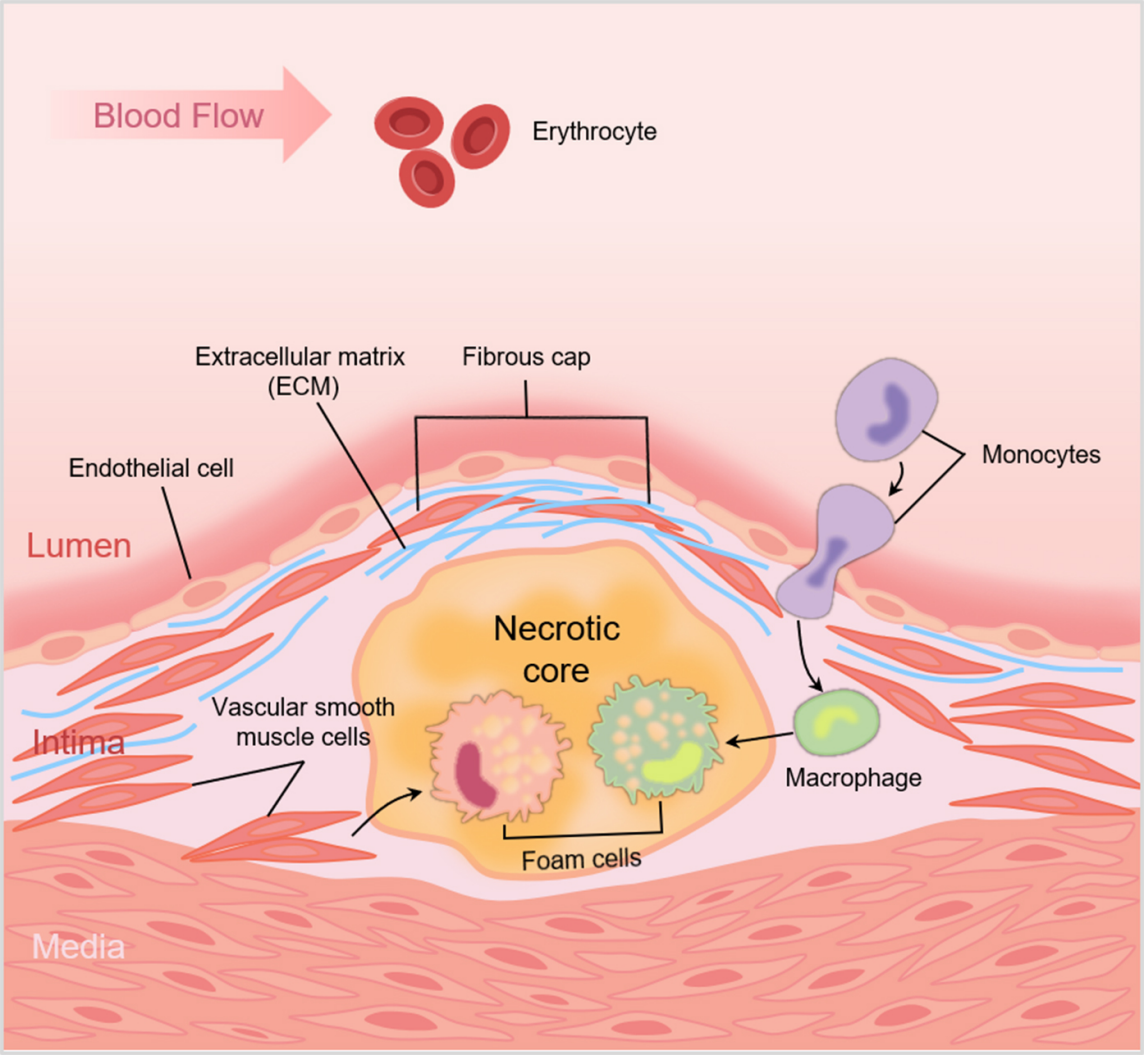
**Schematic illustrating the role of vascular smooth muscle cells (VSMCs) in atherosclerotic plaques**. VSMCs shift from the media to the intima and proliferate; endothelial cells, migrated VSMCs and extracellular matrix secreted by VSMCs together form the fibrous cap. VSMCs absorb lipids to become foam cells, and along with foam cells derived from macrophages, form a necrotic core. Created by Figdraw.

The subcutaneous retention of apolipoprotein B is the initial event of AS. The subcutaneous extracellular matrix, particularly proteoglycans secreted by VSMCs, interacts with lipoproteins, especially low-density lipoproteins (LDL) and lipoprotein (a), to form atherogenic lipoproteins. During this process, secreted phospholipase A2 enzyme hydrolyzes the phospholipids of LDL in the intima of the artery. The hydrolysis of LDL by secreted phospholipase A2 enzyme increases the binding strength of the hydrolyzed LDL with extracellular proteosaccharides, thus, increasing the retention of LDL in the intima of the artery and increasing the possibility of LDL becoming more extensively modified [5]. Subsequently, cells absorb the lipids deposited within the subendothelial space of blood vessels, disrupting lipid metabolism and leading to the formation of foam cells. Traditionally, foam cells in AS were believed to originate from macrophages; however, at least 50% of the foam cells in atherosclerotic plagues are derived from VSMCs rather than macrophages [6]. VSMC-derived foam cell formation is an important marker of AS, particularly in the late stages. Similar to the action of macrophages, increased cholesterol intake and the reduction of cholesterol outflow in VSMCs can lead to foam cell formation [7]. During the pathogenesis of AS, clones of VSMCs migrate from the tunica media layer of the arterial wall to the intima or subendothelium and continue to proliferate, recruiting lipids and changing to a foam cell phenotype, eventually forming foam cells [8, 9] (Fig. 1).

### 2.2 Factors Influencing VSMC-derived Foam Cell Formation

The foam cells derived from VSMCs are formed via the endocytosis of atherosclerotic lipoproteins, primarily modified LDL. Receptor-mediated processes are considered the main pathways for lipid uptake by VSMCs. VSMCs manifest a diverse array of receptors that partake in lipid uptake, which can be categorized into two distinct families, the low-density lipoprotein receptor (LDLR) family and the scavenger receptor (SR) family. The presence of these receptors within VSMCs plays a pivotal role in facilitating the generation of foam cells during the progression of AS [5].

The LDLR family is of great significance in maintaining lipid homeostasis in VSMCs and the pathogenesis of AS, mainly through mediating the removal of cholesterol-containing lipoprotein particles from the circulation. LDLR family members, including low-density lipoprotein receptor-related protein 1 (LRP1), very low-density lipoprotein receptor (VLDLR), and LDLR, participate in lipid uptake by VSMCs during the development of AS [10]. LRP1 exhibits high-level expression in VSMCs and macrophages, while endothelial cells show relatively low expression of LRP1 [11]. LRP1 has a distinct function in the generation of foam cells originating from VSMCs, since it facilitates the *in-vitro* uptake of aggregated LDL (ag-LDL). The K unit CR9 within the LRP1 structure is essential for the binding and internalization of ag-LDL in human VSMCs [12]. Activation of P2Y2 receptors by FLN-A-mediated cytoskeletal rearrangement significantly increases the expression of LRP1 and the uptake of ag-LDL in VSMCs derived from mice [13].

The primary function of the SR family is to eliminate invading lipoproteins, apoptotic cells, and pathogens. SRs are primarily involved in the recognition and internalization of modified LDL, with each SR exhibiting varying binding affinities and preferences for different forms of modified LDL. Both *in vitro* and *in vivo* experiments have shown that the levels of SRs expression in VSMCs mediates the internalization of various modified lipoproteins, such as scavenger receptor class A (SR-A), CD36, and lectin-like oxidized low-density lipoprotein receptor-1 (LOX-1) [14]. SR-A is highly expressed in macrophages, where it mediates lipid uptake, and excess lipid accumulation results in the generation of foam cells [15]. VSMCs and SR-A expression have been detected in atherosclerotic-lesion intima; however, SR-A cannot be detected in normal VSMCs located in the tunica media of arteries. This observation suggests that SR-A activity is upregulated in VSMCs in atherosclerotic lesions [16].

A study has shown that under the influence of transforming growth factor-β (TGF-β), bone marrow mesenchymal stem cells (BMSCs) differentiate into VSMCs. Furthermore, when stimulated by oxidized low-density lipoprotein (ox-LDL), these differentiated VSMCs upregulate the manifestation of SR-A, thereby inducing the conversion of VSMCs to foam cells [17]. The growth arrest-specific (GAS) 6 induced SR-A expression is controlled via the phosphatidylinositol 3-OH kinase (PI 3-kinase)-serine/threonine kinase [Akt/protein kinase B (PKB)] axis and a constitutively active form of Akt enhanced SR-A promoter activity, leading to foam cell formation in human VSMCs [18]. CD36, alternatively referred to as scavenger receptor B2, represents a multifunctional receptor which exhibits extensive expression in diverse cell types, primarily involved in intracellular and extracellular signal transduction, inflammatory responses, and the modulation of lipid metabolism [19]. Increased expression of CD36 can be found in VSMCs within human blood vessels presenting with intimal hyperplasia and obstructive lesions [20]. Triggering receptor expressed on myeloid cells 2 (TREM2) exacerbates lipid influx by upregulating the exhibition of scavenger receptor CD36, promoting the constitution of foam cells derived from VSMCs and macrophages, and thereby exacerbating the development of AS [21]. Titanium dioxide nanoparticles induce the transformation of VSMCs into foam cells *in vitro* as well as *in vivo* by upregulating the expression of CD36 through increased nuclear factor kappa B subunit 2 (NFKB2) [22]. LOX-1, belonging to the E class of SR family, is a transmembrane glycoprotein capable of binding and internalizing ox-LDL in processes involving lipid uptake [23]. c-Fos and enzyme-modified low-density lipoprotein (ELDL) in VSMCs are critical regulatory factors in lipid metabolism, significantly promoting the manifestation of LOX-1, thereby enhancing ox-LDL uptake and inducing the generation of foam cells originating from VSMCs [24, 25]. Macropinocytosis promotes arterial foam cell formation and AS [26]. *In vitro* experiments have found that ELDL endocytosis is mediated by calcium-dependent micropinocytosis, and ELDL upregulates the manifestation of LOX-1 in VSMCs through the receptor for advanced glycation end products (RAGE), thereby promoting ox-LDL uptake and foam cell formation [24]. The results from a mouse model experiment suggest that liver kinase B1 (LKB1) phosphorylation activation of SIRT6 can downregulate the manifestation of LOX-1 in VSMCs, thereby inhibiting foam cell formation and AS [27]. Additionally, ox-LDL-induced hepatoma-derived growth factor (HDGF) upregulates LOX-1 and CD36 expression in aortic VSMCs, promoting foam cell formation [28]. Transmembrane 6 superfamily 2 (TM6SF2) may reduce lipid accumulation in human VSMCs stimulated by ox-LDL by reducing the manifestation of CD36 and LOX-1 [29].

Numerous factors influence the lipid metabolism of VSMCs and are linked to the generation of foam cells originating from VSMCs. P2Y12 is primarily expressed on platelets, with relatively low expression observed in other tissues and cells. In advanced atherosclerosis, activation of the P2Y12 purinergic receptor diminishes cholesterol outflow and fosters the development of foam cells originating from VSMCs by repressing autophagy [30].

**Fig. 2.**
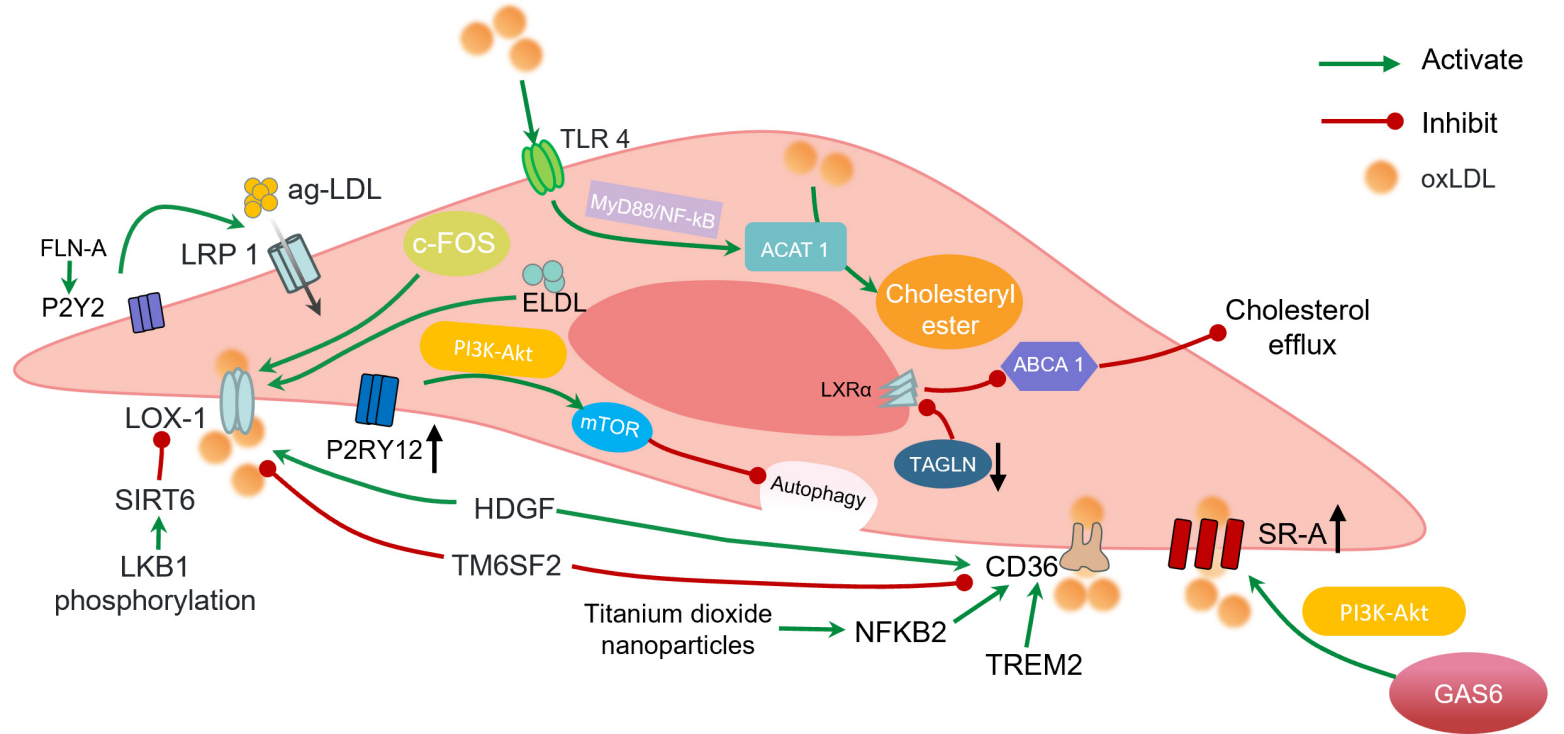
**Factors influencing vascular smooth muscle foam cell formation in atherogenesis**. Green arrows indicate promotion, and red lines indicate inhibition. The upward-directed black arrow denotes upregulation, and the downward-directed black arrow denotes downregulation. SR-A, scavenger receptor class A; LOX-1, lectin-like oxidized low-density lipoprotein receptor-1; mTOR; mammalian target of rapamycin; TLR4, Toll-like receptor 4; ACAT1, A cholesterol acyltransferase 1; ABCA1, ATP-binding cassette transporter A1; NF-κB, nuclear transcription factor kappa B. Created by Figdraw.

Acyl coenzyme A cholesterol acyltransferase 1 (ACAT1) is present in various cells and tissues, including VSMCs [31]. ACAT1 facilitates the formation of foam cell formation by converting free intracellular cholesterol to cholesteryl esters [32], with its expression upregulated by inflammation [33]. Ox-LDL activates Toll-like receptor 4 (TLR4) in VSMCs and upregulates ACAT1 expression via the TLR4/myeloid differentiation primary response 88(MyD88)/nuclear transcription factor kappa B (NF-κB) inflammatory signaling pathway, ultimately promoting the formation of VSMC-derived foam cells [34]. Additionally, murine transgelin (TAGLN) deficiency significantly contributes to VSMC cholesterol accumulation and AS development in mice. The deletion of TAGLN inhibits the nuclear receptor liver X receptor alpha (LXRα) and reduces adenosine triphosphate (ATP)-binding cassette transporter A1 (ABCA1)-mediated cholesterol outflow by promoting actin stress fiber depolymerization, leading to cholesterol accumulation in VSMCs that gradually transform into foam cells [35]. The F-actin-binding protein, Drebrin, decreases the generation of VSMC-derived foam cells and AS development by limiting VSMC NOX-1 activity while promoting plaque stability [36] (Fig. 2).

### 2.3 Modulators Affecting VSMC-derived Foam Cell Formation

Formononetin: Formononetin is the primary flavonoid constituent obtained from astragalus and exhibits protective effects against cardiovascular diseases [37, 38] and suppressive effects on the development of AS, such as obesity [39, 40]. It can downregulate the expression of SR-A by modulating Kruppel-like factor 4 (KLF4), thus, significantly reducing the cholesterol uptake levels in human VSMCs and inhibiting the generation of VSMC-derived foam cells [16] (Table 1, Ref. [16, 41, 42, 43, 44, 45, 46, 47, 48, 49, 50, 51, 52, 53, 54, 55, 56]).

**Table 1.** **Regulators and mechanisms underlying their activity on VSMCs to inhibit AS**.

Regulators inhibiting AS	Targets	Mechanism of action	Effects on VSMCs	References
Formononetin	SR-A	Upregulation of KLF4 levels to downregulate SR-A expression, reducing the uptake of cholesterol by VSMCs	Inhibition of foam cell formation derived from VSMCs	[16]
17β-estradiol	ABCA1 and ABCG1	Upregulation of ABCA1 and ABCG1 expression in VSMCs via the LXRα-dependent pathway, promoting cholesterol outflow from VSMCs	Inhibition of foam cell formation derived from VSMCs	[41]
Genistein	CD36, LOX-1 and CD68	The tyrosine kinase pathway	Inhibition of foam cell formation derived from VSMC	[42]
	LOX-1	Inhibition of SR-C activation of the L-type calcium channel subunit CACNA1C to downregulate LOX-1	Inhibition of ox-LDL-induced formation of foam cells derived from VSMCs	[43]
Atorvastatin	ANG II	Reversal of ANG II-induced decrease in contractile protein in VSMCs	Promotion of VSMC transition to a contractile phenotype	[44]
	PDGF-BB	Regulation of the Akt/FOXO4 axis to downregulate PDGF-BB		
	DNA	Phosphorylation of HDM2, stabilization of NBS-1, and accelerated phosphorylation of ataxia telangiectasia-mutated and histone H2AX expedite DNA repair, alleviating DNA damage in VSMCs	Inhibition of VSMC apoptosis	[52]
Artemisinin	Proteins related to the contractile phenotype	—	Inhibition of the transition of VSMC phenotype to a dedifferentiated phenotype	[45]
Liraglutide	GLP-1 receptor	Activation of AMPK signaling pathway and induction of cell cycle arrest	Inhibition of ANG II-induced migration and proliferation of VSMCs	[46]
Melatonin	PDGF-BB	Reduce mTOR phosphorylation, block cells in the G0/G1 phase, regulate the expression of cell cycle regulatory proteins, and inhibit PDGF-BB	Inhibit proliferation of rat VSMCs induced by PDGF-BB	[47]
Hibiscus leaf polyphenols	PCNA and cyclinD 1	ECG regulates the cell cycle, downregulating the expression of PCNA and cyclin D1	Inhibit proliferation of VSMCs	[48]
	TNF-α	Inhibit Akt/AP-1 pathway	Inhibit migration and proliferation of VSMCs	[49]
Acarbose	Ras	Inhibit small G protein and PI3K/Akt signaling pathways, downregulate Ras protein levels	Inhibit proliferation and migration of VSMCs	[50]
VX-765	Caspase-1	Inhibit Caspase-1 to reduce ox-LDL-induced activation of the NLRP3 inflammasome	Inhibition of NLRP3 inflammasome-induced apoptosis in VSMCs	[51]
Hydrogen sulfide	caspase-3/9, Bax and Bcl-2	Reduced caspase-3/9 activity and Bax/Bcl-2 ratio in ox-LDL-stimulated VSMCs	Inhibition of VSMCs apoptosis	[52]
6-shogaol	P53	Inhibition of the upregulation of the OXR1-p53 axis	Inhibition of VSMCs apoptosis	[53]
Paeonol	Beclin-1	Activation of the class III PI3K/Beclin-1 signaling pathway to induce autophagy in VSMCs	Induce autophagy in VSMCs and inhibit VSMCs apoptosis	[54]
Celastrol	ROS	Activation of autophagy to reduce the production of ROS	Inhibition of VSMCs senescence	[55]
	ABCA1	Activation of LXRα to upregulate the expression of ABCA1	Inhibition of foam cell formation derived from VSMCs	[56]

ABCA1, ATP-binding cassette transporter A1; ABCG1, ATP-Binding Cassette Sub-Family G Member 1; KLF4, Kruppel-like factor 4; ANG II, angiotensin II; HDM2, human double minute protein 2; NBS-1, Nijmegen breakage syndrome protein; GLP1, glucagon-like peptide-1; OXR1, oxidation resistance protein 1; LDL, low-density lipoproteins; TNF-α, tumor necrosis factor alpha; ROS, reactive oxygen species; PCNA, proliferating cell nuclear antigen; ECG, (-)-Epicatechin gallate.

Estrogen: Estrogen plays a crucial role in preventing the development of AS, which is reflected by the lower incidence of cardiovascular diseases in pre-menopausal females than in males [57, 58]. However, post-menopausal women have a similar incidence of cardiovascular diseases as males [59]. 17β-Estradiol upregulates ABCA1 and ATP-Binding Cassette Sub-Family G Member 1 (ABCG1) expression in VSMCs via the LXRα-dependent pathway, promoting the cholesterol outflow of VSMCs and thus inhibiting the generation of VSMC-derived foam cells [41] (Table 1). Furthermore, estrogen acts on hepatocyte estrogen receptor alpha (LKO-Erα) to increase reverse cholesterol formation, thereby reducing AS [60]. Estrogen acts independently of the LKO-Erα to prevent AS [61].

Genistein: Genistein is a bioactive substance derived from the leguminous plant dyewood (*Genista tinctoria*), known for its promising anticancer potential and effects on the pathophysiological processes related to the metabolic syndrome and obesity [62]. Genistein acts as a cannabinoid receptor 1 antagonist and reduces cannabis-induced A [63]. Additionally, genistein inhibits VSMC-derived foam cell formation by reducing the protein manifestation of CD36, LOX-1, and CD68 via the tyrosine kinase pathway [42] (Table 1). Furthermore, genistein can reduce the absorption of ox-LDL by downregulating LOX-1, following the inhibition of the L-Ca channel subunit, calcium voltage-gated channel subunit alpha 1C (CACNA1C), activated by SR-C [43] (Table 1).

## 3. Phenotypic Switching of VSMCs

### 3.1 Phenotypic Switching of VSMCs During the Development of AS

VSMCs, the main cell type in blood vessel walls and atherosclerotic plaques, have high plasticity. VSMCs are involved in the development of AS by switching to other phenotypes [64]. In the process of AS, VSMC has a variety of different states, and its morphology and function can be altered [65]. VSMC phenotype switching refers to changes in VSMC morphology, structure, and function to adapt to environmental changes in different disease states, which is the basic mechanism for the development of AS. VSMCs play indispensable roles in the maintenance of vessel wall integrity, contractility, and elasticity [66]. In normal blood vessels, VSMCs preserve a resting, contractile state. VSMCs with the contractile phenotype express various unique contractile proteins such as alpha-smooth muscle actin (ACTA2), TAGLN, Calponin 1, and Myosin Heavy Chain 11 (MYH11), which rarely proliferate or migrate [67]. In AS, VSMCs gradually lose their contractile markers, de-differentiate into synthetic phenotypes of excessive proliferation and migration, and trans-differentiate into macrophage- and osteoblast-like cells, which are involved in the development of atherosclerotic plaques [67]. Studies on lineage tracing have demonstrated that the proportion of cells in atherosclerotic plaques originating from VSMCs ranges from 30% to 70%. Therefore, modulating the VSMC phenotype and de-differentiation are potential therapeutic strategies for treating AS [9, 68]. Inhibiting the transition from the VSMC phenotype to the de-differentiated phenotype has a protective atherogenic effect [9, 69]. The importance of the role of VSMC phenotypic switching in the development of atherosclerotic plaques has led to an increasing number of studies on the modulation of VSMC phenotypic switching.

### 3.2 Factors and Mechanisms Affecting the Phenotypic Switching of VSMCs

**Fig. 3.**
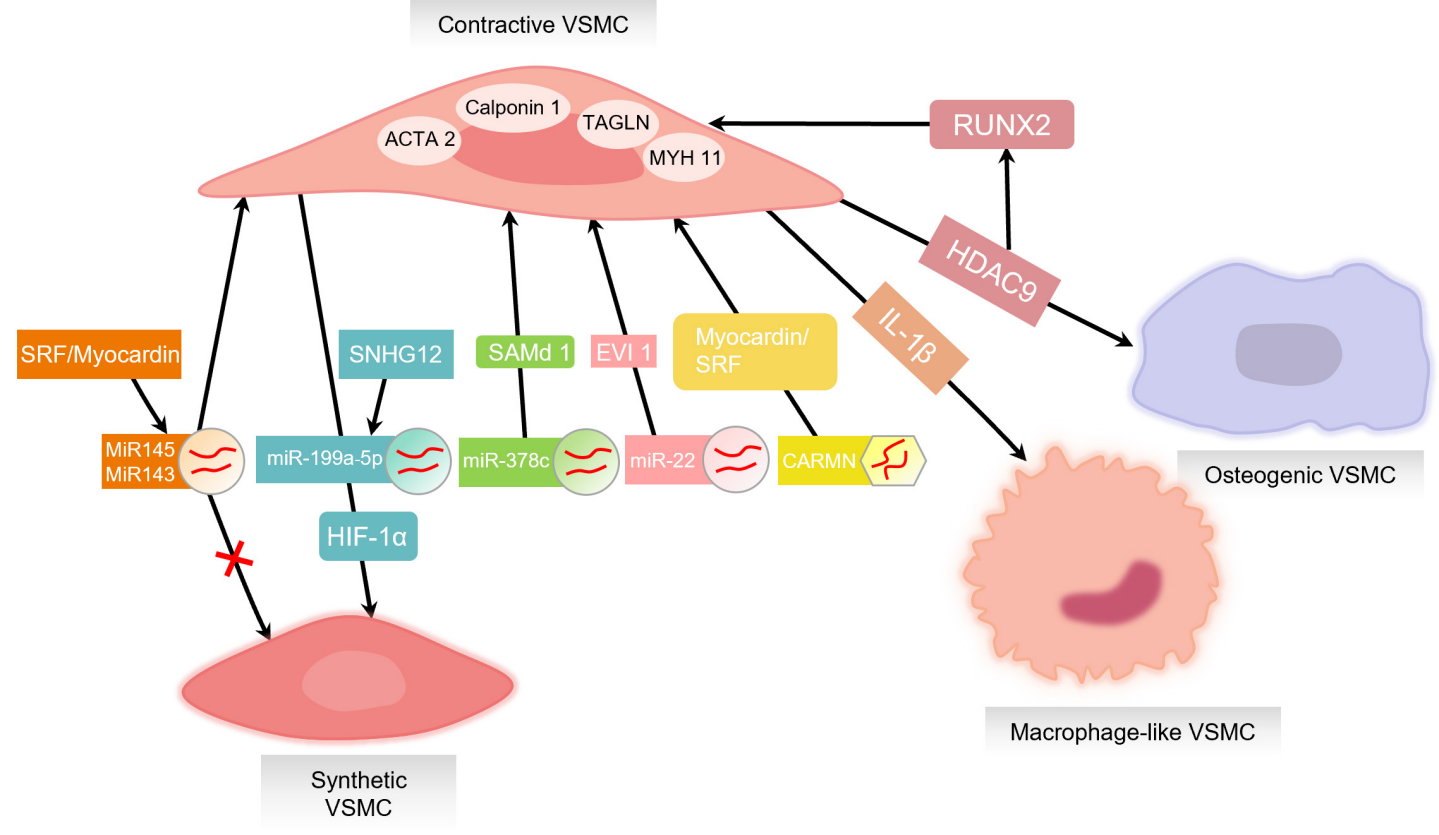
**Factors influencing VSMC phenotype switching**. Contractive VSMCs express various unique contractile proteins, such as alpha-smooth muscle actin (ACTA2), transgelin (TAGLN), Calponin 1, and MYH11. MicroRNAs (miRNAs) have an important function in the control of VSMC phenotype. miRNA-143 and miRNA-145 promote the maintenance of the contractive phenotype in VSMC’s and inhibit conversion to synthetic VSMCs. miRNA-143 and miRNA-145 expressions are positively regulated by SRF/Myocardin complexes. The SNHG12 acts on miR-199 a-5 p/hypoxia inducible factor-1 (HIF-1α) to promote the conversion from a contractile to a synthetic phenotype. MiR-378c directly targets the predicted transcriptional repressor sterile alpha motif domain 1 (SAMd 1), miR-22 targets ecotropic virus integration site 1 protein homolog (EVI 1), and lncRNA CARMN acts with myocardin and SRF. These factors promote VSMCs to maintain a contractile phenotype and prevent AS. Interleukin (IL)-1β is a key transcription factor mediating VSMC transition to a macrophage-like phenotype during the progression of AS. Histone deacetylase 9 (HDAC9) gene expression promotes an osteoblastic VSMC phenotype that enhances calcification and reduces contractility. Inhibition of HDAC9 activity decreases runt-related transcription factor 2 (RUNX2) expression, favoring an increased contractive VSMC phenotype thereby leading to decreased proliferation and calcification. Created by Figdraw.

In the field of epigenetics, the functions of microRNAs (miRNAs) and long non-coding RNAs (lncRNAs) in regulating the VSMC phenotype have primarily been described in the development of AS. miRNAs are regulators of vascular diseases, and play a crucial role in the regulation of the VSMC phenotype [70]. For example, in the vascular system, miR-145 is the most expressed miRNA. It ensures the maintenance of the VSMC contractile phenotype by facilitating the upregulation of contractile genes and proteins, among which are cardiac proteins, calmodulin, and ACTA2, while downregulating the expression of synthetic genes [71]. miRNA-143/145 acts synergistically on multiple transcription factors, such as KLF4, myocardin, and erythroblast transformation specific (ETS) domain protein-1 (ELK-1, ETS oncogene family member), to promote VSMC differentiation and inhibit proliferation. The expression of miRNA-143 and miRNA-145 is targeted by serum response factor (SRF), cardiac erythropoietin, and NK2 transcription factor-related, site 5 (Nkx2-5) [71]. Myocardin and SRF complexes increase VSMC differentiation marker expression by binding to the CArG box of the target promoters [72]. Vengrenyuk *et al*. [73] found that maintaining the expression of myocardin or miR-143/145 could prevent or reverse the change of VSMC from a contractile phenotype to a macrophage-like phenotype induced by an increased cholesterol load. The stem cell pluripotency genes *KLF4* and octamer-binding transcription factor 4 (Oct4) are essential for the pathogenesis of advanced atherosclerotic lesions via the regulation of VSMC phenotypic changes [8]. Chin *et al*. [70] used nanoparticle-mediated miR-145 expression to inhibit plaque-proliferating cell types derived from VSMCs by promoting the contractile VSMC phenotype. Small nucleolar RNA host gene 12 (SNHG12) acts on miR-199 a-5 p/hypoxia inducible factor-1 (HIF-1α) to participate in regulating the phenotype of VSMCs [74]. Inhibition of miR-378c expression promotes phenotypic transition in VSMCs, with miR-378c directly targeting the predicted transcriptional repressor sterile alpha motif domain 1 (SAMd 1) to prevent AS [75]. miRNA-22 is as a newly discovered agent for regulating phenotypic changes and neointima development in VSMCs. In murine and human femoral artery disease models, the ecotropic virus integration site 1 protein homolog (EVI 1), which acts as a novel target of miR-22, determines the VSMC phenotype as well as injury-triggered arterial remodeling [76]. Bell *et al*. [77] found that smooth muscle and endothelial cell-enriched migration/differentiation-associated long noncoding RNA (SENCR), a lncRNA enriched in smooth muscle and endothelial cells and related to migration/differentiation, is a novel type of cytopasmic vascular cell. Its role appears to be safeguarding the contractile phenotype of smooth muscle cells. Cardiac mesoderm enhancer-associated non-coding RNA (CARMN) is a highly abundant smooth muscle cell-specific lncRNA that can preserve the contractile state of VSMCs by physically binding to the key transcriptional cofactor myocardin and can also regulate the VSMC phenotype through direct engagement with the VSMC transcription factor SRF, which is a crucial regulator of VSMC proliferation and differentiation [78] (Fig. 3).

The methyltransferase disruptor of telomeric silencing 1-like (DOT1L) is involved in the epigenetic control of VSMC gene expression, and DOT1L promotes AS development by directly regulating the NF-κB pathway to regulate the expression of the inflammatory cytokines CCL5 and CXCL10 [79]. Histone deacetylase 9 (HDAC9) gene expression promotes the osteoblast-like VSMC phenotype, enhances calcification, and reduces contractility, whereas the inhibition of HDAC9 activity decreases runt-related transcription factor 2 (RUNX2) expression, favoring an increased contractile VSMC phenotype and reducing proliferation and calcification [80]. The NLR family pyrin domain containing 3 (NLRP3), a powerful mediator of the inflammatory response, promotes lipid deposition, foam cell accumulation, and AS progression by releasing the pro-inflammatory mediators IL-1β and IL-18 [81]. IL-1β is an important transcription factor mediating the dedifferentiation of VSMCs into a macrophage-like phenotype during AS progression [82]. Rong *et al*. [83] demonstrated that with cholesterol loading in VSMC, phenotypic changes are under the control of mRNA level regulation and end up in a trans-differentiation resembling a macrophage-like state. VSMCs in plaques are activated and de-differentiated into “inflammatory” macrophage-like cells expressing clusters of differentiation 68 (CD68) (CD68+VSMCs), which produce extracellular traps similar to macrophages and are involved in regulating the de-differentiation direction of VSMCs via the STING-SOCS1 or TLR4 signaling pathway. CD68+VSMCs are the primary sources of macrophages in advanced atherosclerotic plaques [84] (Fig. 3).

### 3.3 Modulators Affecting Phenotypic Switching in VSMCs

Atorvastatin: As a 3-hydroxy-3-methylglutaryl coenzyme A reductase inhibitor, atorvastatin is routinely used to regulate blood lipid levels and has anti-inflammatory effects [85]. Atorvastatin increases VSMC contractile protein expression (e.g., ACTA2, MYH11, and TAGLN) and can reverse the effect of angiotensin II (ANG II) in VSMCs. Thus, atorvastatin can regulate ANG II—associated VSMC phenotypic switching via the epigenetic regulation of contractile protein expression. Additionally, Atorvastatin may attenuate platelet-derived growth factor (PDGF)-BB-induced VSMC phenotype transition by modulating the Akt/forkhead box O (FOXO) 4 axis, thereby alleviating abnormal proliferation and migration of VSMCs [44] (Table 1).

Prostaglandins: Tumor necrosis factor alpha (TNFα) is an important inflammatory cytokine during AS progression and is involved in vascular remodeling [86]. TNFα induces cyclooxygenase-2 (COX2) expression in various cells, which in turn regulates thromboxane A2 (TXA2) and mediates the production of prostaglandins, such as prostaglandin D2 (PGD2), prostaglandin I2 (PGI2), prostaglandin E2 (PGE2), and prostaglandin F2 alpha (PGF2α) [87]. In this review, we have concentrated on the effects of the first two factors in VSMC phenotypic transition. PGD2 may mediate peroxisome proliferator-activated receptor delta (PPARδ) activation and promote dedifferentiation of the VSMC contractile phenotype into a synthetic phenotype [88]. Additionally, PGI2 is indirectly involved in VSMC phenotypic alteration. PGI2 is used as a vasodilator, platelet aggregation inhibitor, and VSMC proliferation inhibitor [89]. The cardioprotective effects of PGI2 are mediated by the prostacyclin receptor (IP receptor) via the adenylate cyclase/secondary messenger cyclic phosphate adenosine/protein kinase A signaling pathway [90]. In the absence of thromboxane-prostanoid receptors, PGI2/iloprost treatment stimulates cell proliferation, upregulates synthetic proteins, downregulates contractile proteins, and promotes VSMC phenotypic switching [91].

Artemisinin (ART): ART is a sesquiterpene lactone endoperoxide extracted by Youyou Tu from the plant Artemisia annua [92], which has a potent anti-malarial effect [93]. Research has shown the therapeutic benefits of ART in many other diseases. Du *et al*. [45] investigated whether ART has anti-atherosclerotic effects through *in vivo* and *in vitro* experiments. ART substantially decreased plaque areas and increased the levels of contractile phenotype-related proteins in *ApoE*^-⁣/-^ mice, reducing atherosclerotic lesions by inhibiting the transition of the VSMC phenotype to a de-differentiated phenotype (Table 1).

Retinoids: Vitamin A, also known as retinol, has natural and synthetic derivatives called retinoids. It is an essential vitamin for the body. To obtain it, one has to consume vitamin A-rich foods and foods containing the carotenoid β-carotene, which is comprised of two retinol molecules. The most representative is all-trans retinoic acid (atRA), atRA was found to promote cell differentiation *in vitro*, therefore, atRA and other natural and synthetic retinoic acid (RA) have been widely used to treat cancer. Perturbation in differentiation and growth is a common mechanism of cancer and cardiovascular diseases. It has been found that atRA can promote VSMC differentiation and maintain systolic phenotypes [94]. Pan *et al*. [95] discovered a new cell state in the process of smooth muscle cell phenotypic transformation by means of single-cell genomics. The VSMC-derived intermediate cells, named “SEM” cells, were pluripotent and had the ability to differentiate into macrophage-like and fibrochondrocyte-like cells, and also revert back to the VSMC phenotype. They found that RA is an important regulator of this process. The initiation of RA signaling via atRA decreased the transformation of VSMC into SEM cells, decreased the atherosclerotic load, and enhanced the stability of the fibrous cap.

## 4. Proliferation and Migration of VSMCs

### 4.1 Proliferation and Migration of VSMCs During the Development of AS

VSMCs promote their own migration and proliferation via phenotypic switching in AS. When diffuse intimal thickening occurs early in a lesion, VSMCs migrate from the media to the intima and transform from a quiescent contractile phenotype to an activated synthetic phenotype [96]. Growth factors, receptors of synthetic VSMCs, and extracellular matrix proteases are upregulated, contractile protein expression is reduced, and results in migration and proliferation from the media to the intima. These factors are involved in the formation of the initial atherosclerotic plaques. In the late stage of AS, synthetic VSMCs accumulate and further proliferate, forming a fibrous cap to stabilize atherosclerotic plaques by protecting them from rupture. This protection is beneficial in slowing the formation of advanced atherosclerotic lesions to some extent [97]. The regulatory process of VSMC proliferation and migration is extremely complex and involves multiple regulatory molecules and signaling pathways that are involved in the entire process of AS formation.

### 4.2 Factors and Mechanisms Affecting the Proliferation and Migration of VSMCs

**Fig. 4.**
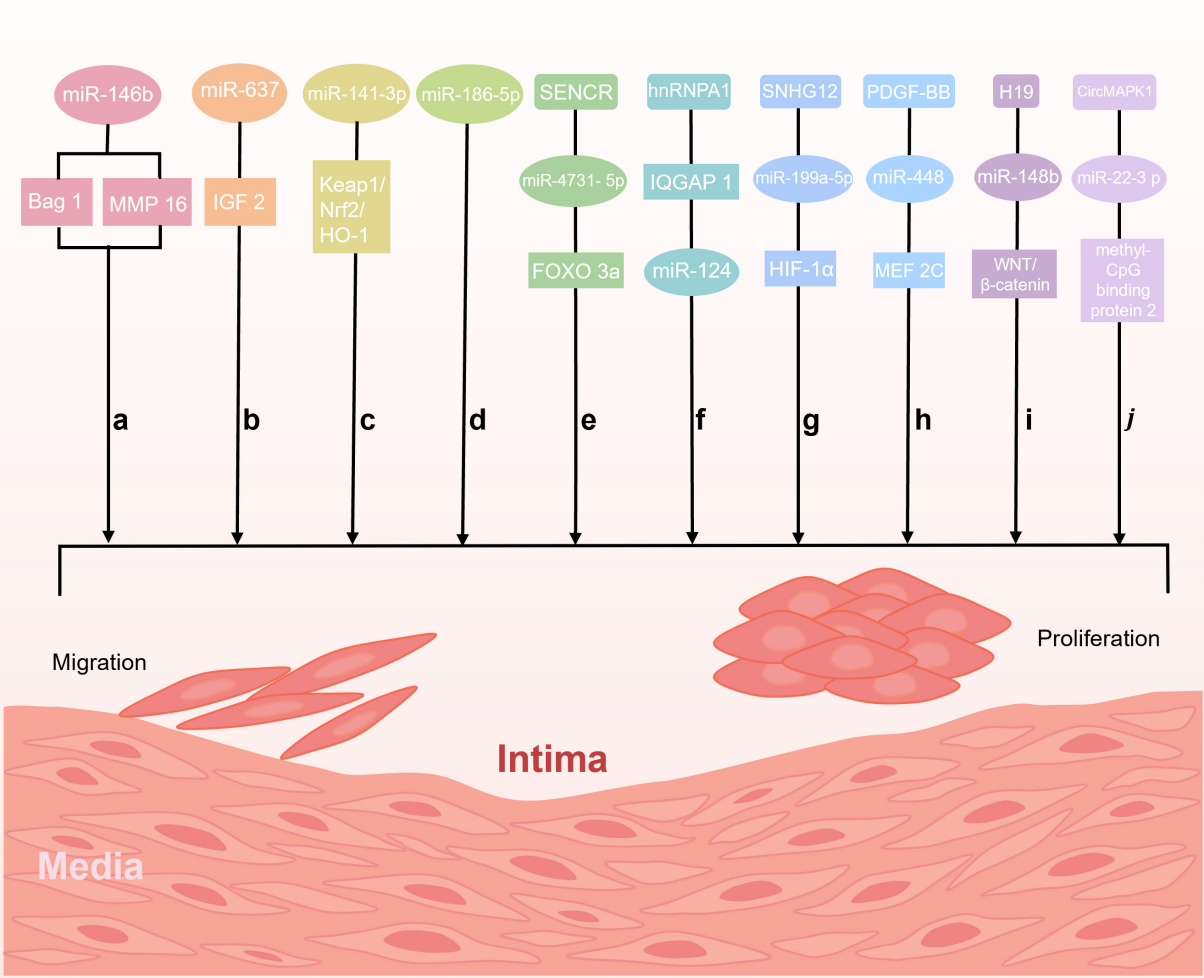
**MiRNAs regulate the proliferation and migration of VSMCs**. (a) miR-146 b overexpression inhibits the proliferation and migration of VSMCs via the downregulation of Bag 1 and matrix metalloproteinase-16 (MMP-16). (b) miR-637 inhibits proliferation and migration of VSMCs by regulating insulin-like growth factor-2 (IGF2). (c) miR-141-3 p inhibits proliferation and migration of VSMCs by regulating the Keap1/Nrf 2/HO-1 pathway. (d) Downregulation of miR-186-5p inhibits the proliferation and migration of VSMCs. (e) SENCR influence the proliferation and migration of VSMCs by regulating the miR-4731-5 p/forkhead box O (FOXO) 3a pathway. (f) Heterogeneous nuclear ribonucleoprotein A1 (hnRNPA1) targets the regulation of IQ motif-containing guanosine triphosphate (GTP)ase activating protein 1 (IQGAP 1) to reduce VSMC proliferation and migration via the upregulation of miR-124 expression. (g) SNHG12 targets the miR-199a-5p/HIF-1α axis to promote the proliferation and migration of VSMCs. (h) Platelet-derived growth factor (PDGF)-BB induces miR-448 expression, which in turn targets myocyte-specific enhancer factor 2C (MEF2C), acting on VSMC proliferation and migration. (i) H19 inhibits miR-148b expression and thus activates the WNT/β-catenin signaling pathway to promote proliferation and migration of VSMCs. (j) Circular mitogen-activated protein kinase (circMAPK 1) regulates miR-22-3p/Methyl-CpG binding protein 2 axis to promote the proliferation and migration of VSMCs. Created by Figdraw.

miRNAs are short, endogenous, non-coding RNAs comprising ~22–24 nucleotides. To date, thousands of miRNAs have been identified, most of which play key regulatory roles in animals, and are among the most prominent regulators of gene expression. miRNAs also have critical regulatory roles in VSMC proliferation and migration; for example, miR-146b overexpression inhibits the proliferation and migration of VSMCs by downregulating Bag-1 and matrix metalloproteinase (MMP)-16 [98], miR-637 inhibits VSMC proliferation and migration by regulating insulin-like growth factor-2 [99], and miR-141-3 p inhibits the proliferation and migration of VSMCs by regulating the Keap1/Nrf 2/HO-1 pathway [100]. Additionally, miR-186-5p acts as an inhibitor of VSMC proliferation and migration when it is downregulated [101]. miRNAs also mediate VSMC proliferation and migration as intermediate factors. SENCR influences VSMC proliferation and migration by regulating the miR-4731-5 p/FOXO 3a pathway [102]. Furthermore, heterogeneous nuclear ribonucleoprotein A1 (hnRNPA1) targets regulation of the IQ motif-containing GTPase activating protein 1 (IQGAP 1) to reduce VSMC proliferation and migration by upregulating miR-124 [103]. Circular RNA mitochondrial translation optimization (CircMTO) 1 inhibits ox-LDL stimulation-induced VSMC proliferation by regulating the miR-182-5 p/RASA 1 axis [104]. In contrast, SNHG12 targets the miR-199 a-5 p/HIF-1α axis [74]. PDGF-BB induces miR-448 expression, which in turn targets myocyte enhancer factor 2C (MEF2C), thereby acting on VSMC proliferation and migration [105], H19 inhibits miR-148b expression and thus activates the Wnt/β-catenin signaling pathway [106], and circular mitogen-activated protein kinase (CircMAPK) 1 regulates miR-22-3 p/Methyl-CpG binding protein 2 axes [107]. These factors and genes act on different miRNAs and promote VSMCs to proliferate and migrate via different signaling pathways (Fig. 4).

In addition to these miRNAs, numerous other proteins and related factors have major roles in VSMC proliferation and migration. KLF4 represents a vital component that inhibits VSMC proliferation. C/EBP homologous protein (CHOP) deficiency in aortic VSMCs increases KLF4 expression via the endoplasmic reticulum stress effector activating transcription factor 4 (ATF4), thereby reducing proliferation [108]. Omentin-1, a novel adipocytokine expressed in visceral adipose tissue, markedly inhibits ANG II–induced human VSMC migration and PDGF-BB-induced human VSMC proliferation [109]. Insulin-like growth factor 1 (IGF1), a major autocrine/paracrine growth factor, promotes proliferation, migration, and survival of VSMCs in the same manner, and these effects are associated with IGF1-induced disruption of Akt signaling [110]. Nuclear factor erythroid 2 related factor 2 (NRF2), a crucial antioxidizing factor, reduces the atherosclerotic plaque load and the proliferation and migration of VSMCs as the downregulation of NRF2 inhibits LOX-1 expression. Regarding proteins, myeloid-derived CHOP promotes VSMC proliferation by downregulating KLF4 [108], and CD98 deletion reduces VSMC proliferation and migration [111]. CD73 promotes AS by promoting VSMC migration, proliferation, and foam cell transformation as well as increasing lipid levels [112]; chromobox protein homolog 3 (CBX3) regulates VSMC contraction and collagen gene expression via the NOTCH3 pathway while inhibiting VSMC proliferation and migration [113].

### 4.3 Modulators Affecting VSMC Proliferation and Migration

Liraglutide: Liraglutide is an analog of glucagon-like peptide-1 (GLP1), an intestinal proinsulin hormone secreted by intestinal L cells after eating that stimulates insulin secretion and inhibits glucagon secretion in a glucose-dependent manner to control postprandial blood glucose [114]. GLP-1 and GLP-1 receptor agonists have independent roles in the cardiovascular system and can improve endothelial and left ventricular function [115]. Liraglutide, a long-acting GLP-1 receptor agonist [116], can impede the ANG II-elicited migration and proliferation of VSMCs by turning on adenosine monophosphate-activated protein kinase signaling and prompting cell cycle arrest, consequently retarding the progression of AS [46] (Table 1).

Melatonin: Melatonin is composed of N-acetyl-5-methoxytryptamine, an indoleamine that aids in sleep, regulates circadian rhythms [117], and inhibits tumors and inflammation [118, 119]. Melatonin has multiple roles in the cardiovascular system, such as preventing endothelial scorch formation [120], reducing endothelium-derived adhesion molecule formation [121], regulating cholesterol [122], and inhibiting LDL oxidation [123]. In 2019, Li *et al*. [47] proposed that melatonin inhibited the proliferation of VSMCs triggered by PDGF-BB in rats by reducing phosphorylation of the mammalian target of rapamycin (mTOR) protein, blocking cells in the G0/G1 phase, and regulating the expression of cell cycle regulatory proteins (Table 1).

Hibiscus leaf polyphenols: Plant polyphenols possess a diverse range of pharmacological and biological activities, including antioxidative, anti-inflammatory, anti-hypolipidemic, and anti-cancer effects [124]. Hibiscus leaf polyphenols are plant polyphenols extracted from Hibiscus leaves, which are an edible part of Hibiscus plants [125]; the leaves have anti-inflammatory and antioxidant properties [126]. (-)-Epicatechin gallate has the highest contents among hibiscus leaf polyphenols and plays an antiproliferative role by regulating the cell cycle and downregulating the manifestation of proliferating cell nuclear antigen as well as cyclin D 1 [48] (Table 1). Chou *et al*. [49] found that hibiscus leaf polyphenols abrogated TNFα-induced matrix MMP-9 expression and migration and proliferation of VSMCs by inhibiting the PKB (also known as Akt)/activator protein-1 (AP-1) pathway (Table 1).

In addition to the aforementioned modulators, acarbose and nicotine mediate VSMC proliferation, and migration. Acarbose is a well-known drug for treating diabetes; however, in 2018, acarbose was discovered to inhibit the proliferation and migration of RasG 12 V A7 r5 cells (k-RasG 12 V-transfected VSMCs) by blocking small G protein and PI3K/Akt signaling pathways [50] (Table 1). Exosomal miR-21-3p from nicotine-treated macrophages may speed up atherosclerosis by promoting VSMC migration and proliferation via phosphatase and tension homologue [127].

## 5. Apoptosis of VSMCs

### 5.1 Apoptosis of VSMCs During the Development of AS

VSMCs are involved in AS development and have a key role in maintaining the stability of advanced atherosclerotic plaques. The main pathological change in advanced AS is the formation of fibrous atheromatous plaques (composed of necrotic cores and fibrous caps). The fibrous caps comprise proliferating VSMCs and collagen-rich extracellular matrix [128]. VSMCs secrete extracellular matrix and form protective fibrous caps over advanced atherosclerotic lesions, which maintain plaques, prevent rupture, and reduce clinical atherothrombosis and acute cardiovascular and cerebrovascular events [129]. Apoptosis of VSMCs mediates the progression of late AS development and plaque changes, thereby influencing changes in atherosclerotic plaque fibrous cap thickness and matrix composition. In 2006, Clarke *et al*. [130] found that VSMC apoptosis alone was sufficient to induce atherosclerotic plaques to become fragile and prone to rupture. Therefore, the survival of VSMCs is critical for the stability of advanced plaques, and the modulation of VSMC apoptosis may become an important therapeutic intervention to limit the progression of AS.

### 5.2 Factors and Mechanisms Affecting Apoptosis of VSMCs

**Fig. 5.**
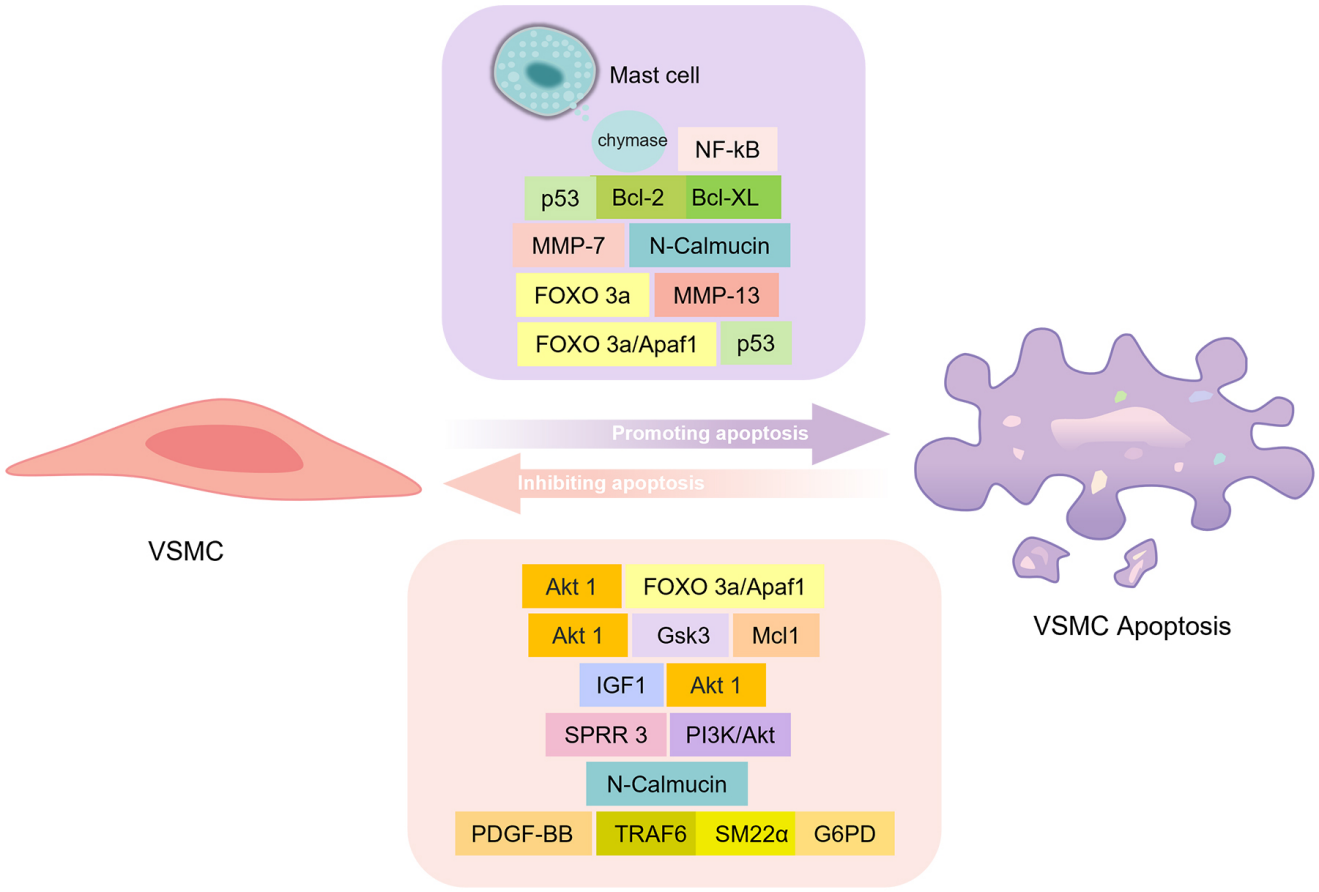
**Factors influencing VSMC apoptosis and their signal pathways**. The purple box shows the available factors and mechanistic pathways in the promotion of VSMC apoptosis. Apoptotic protease activating factor 1 (Apaf 1) is a forkhead box (FOXO) 3a transcriptional target, and Apaf 1 induces VSMC apoptosis and can be transcriptionally induced by apoptotic effectors such as p53. Matrix metalloproteinase 13 (MMP-13) is a direct transcriptional target of FOXO 3a in VSMCs, and FOXO 3a substantially triggers the generation and release of MMP-13 by VSMCs and promotes apoptosis. MMP-7 is involved in the cleavage of N-calmucin and thus promotes VSMC apoptosis. p53 often forms complexes that possess inhibitory functions and involve Bcl-XL and Bcl-2 to promote apoptosis via the mitochondrial pathway. Mast cells release chymase in an autocrine manner, and chymase induces VSMC apoptosis by blocking nuclear transcription factor kappa B (NF-κB)-mediated survival signals. The pink box shows the multiple factors and mechanistic pathways involved in the inhibition of VSMC apoptosis. AKT1 primarily targets FOXO 3a/Apaf 1 to promote VSMC survival in atherosclerotic lesions. Additionally, AKT1 regulates GSK-3 phosphorylation and decreases VSMC apoptosis by means of phosphorylation and the following ubiquitin-mediated breakdown of Mcl1, a member of the Bcl-2 family, within VSMCs. Insulin-like growth factor-1 (IGF1) works against VSMC apoptosis via AKT1. Activation of Akt by small proline-rich repeat protein (SPRR3) achieves a protective effect in reducing VSMC apoptosis. N-Calmucin can reduce VSMC apoptosis by mediating intercellular contacts. In response to PDGF-BB stimulation, TNF receptor associated factor 6 (TRAF6) mediates TAGLN ubiquitination and subsequently induces elevated levels of activated glucose-6-phosphate dehydrogenase (G6PD) that stimulates increased nicotinamide adenine dinucleotide phosphate (NADPH) production, thereby enhancing VSMC viability and reducing apoptosis via glutathione (GSH) homeostasis. Created by Figdraw.

Serine/threonine kinase Akt (also called protein kinase B or PKB) is a versatile kinase involved in cell growth, metabolism, and survival [131]. It is important in mediating VSMC apoptosis during the development of AS, and AKT1 deletion in VSMCs promotes apoptosis and plaque necrosis [132]. AKT1 promotes VSMC survival in atherosclerotic lesions by primarily targeting FOXO 3a-Apaf 1. Apaf 1 induces VSMC apoptosis, and AKT1 can inhibit Apaf 1 activity, which is the transcriptional target of FOXO 3a and can be transcriptionally induced by apoptotic effectors, such as p53 [133]. AKT1 modulates the phosphorylation of glycogen synthase kinase-3 (GSK-3). This mechanism regulates apoptosis in VSMCs through phosphorylation and then triggering the ubiquitin-mediated degradation of Mcl1, which belongs to the Bcl-2 family [133]. IGF1 is a potent anti-apoptotic cytokine of VSMCs mediated primarily via AKT1 *in vitro*. IGF1 is essential for VSMC survival during oxidative stress [134] and acts by reducing IGF1 receptor expression and signaling in VSMCs [135] and partly inhibiting IGF1 receptor expression via p53 induction [136]. The small proline-rich repeat protein (SPRR3), an atherogenic protective factor in VSMCs, activates Akt to achieve a positive effect in reducing apoptosis. This pathway is independent of the insulin-like growth factor signal pathway [137]. The *p53* gene, which is crucial for tumor suppression, encodes a transcription factor. The activation of several genes involved in growth arrest and apoptosis is achieved through this factor [138, 139]. p53 combines with Bcl-XL and Bcl-2 to create inhibitory complexes, which then promote apoptosis via the mitochondrial pathway [140]. In addition, p53 regulates the sensitivity of apoptosis mediated by death receptors (e.g., Fas/DR5) [141, 142]. p53 expression influences its effects on cells; low p53 expression usually induces growth arrest, whereas high p53 expression induces apoptosis by reducing cell sensitivity [143]. However, p53 may have the opposite effects on apoptosis. Endogenous p53 in VSMCs protects VSMCs from apoptosis, and its protective effect may be partly due to the inhibition of the DNA damage response in VSMCs [144]. The regulation of VSMC survival by the lncRNA *GAS5*, as a negative regulator, occurs in vascular remodeling. Meanwhile, it also modulates VSMC cycle arrest and apoptosis via the p53 pathway [138] (Fig. 5).

N-cadherin, an intercellular adhesion molecule, reduces VSMC apoptosis by reducing intercellular contact in *in vitro* experiments [145]. MMP-7 participates in N-calmodulin cleavage and regulates VSMC apoptosis, which may contribute to the formation and rupture of plaques [146]. MMP-13 is a direct transcriptional target of FOXO 3a in VSMCs. FOXO 3a significantly induces MMP-13 expression and secretion in VSMCs and promotes apoptosis, and MMP-13-specific inhibitors reduce FOXO 3a-mediated apoptosis [147]. Dong *et al*. [148] identified a new mechanism in the link between glucose metabolism and VSMC survival, namely the TRAF6- TAGLN -G6PD pathway. TRAF6 mediates TAGLN ubiquitination under PDGF-BB stimulation, which subsequently induces elevated levels of activated G6PD and stimulates increased nicotinamide adenine dinucleotide phosphate production, thereby enhancing VSMC viability and reducing apoptosis via glutathione. Mast cells are also important mediators of AS development, and activated mast cells may contribute to plaque fragility. The mechanism of promotion of VSMC apoptosis involves the activation of TLR4 on mast cells to induce nuclear translocation of NF-κB, which induces the production of the pro-inflammatory cytokine IL-6. Mast cells subsequently release chymotrypsin in an autocrine manner [149]. Chymotrypsin induces VSMC apoptosis by blocking NF-κB-mediated survival signals [150] (Fig. 5).

### 5.3 Modulators Affecting Apoptosis of VSMCs

VX-765: Caspase-1 is an important factor in apoptosis and mediates the maturation and release of IL-1. In addition, VX-765, a caspase-1 inhibitor, markedly reduces NLRP3 inflammatory vesicle-induced apoptosis activated by ox-LDL in VSMCs *in vitro* while reducing IL-1β production and attenuating the inflammatory response within plaques [51] (Table 1).

Hydrogen sulfide: Hydrogen sulfide (H_2_S), an active ingredient that can be extracted from garlic, is an endogenous gas signal molecule beneficial in the therapy of cardiovascular diseases such as AS [151, 152]. H_2_S may decrease AS progression and prevent adverse cardiovascular events by inhibiting plaque instability. Clinical findings suggest that reduced endogenous H_2_S production may predispose patients with stable coronary artery disease to develop vulnerable plaque rupture and acute coronary syndromes [153]. Xiong *et al*. [52] found that the exogenous H_2_S donor sodium thiosulfate (NaHS) administered daily to *ApoE*^-⁣/-^ mice on a high-fat diet increased plaque collagen and the width of the fibrous cap, lowered lipid levels, and reduced platelet formation. Plaque stability is improved primarily by reducing apoptosis of VSMCs and inhibiting the expression of the collagen-degrading enzyme MMP-9 (Table 1).

Statins: The use of Hydroxymethylglutaryl coenzyme A reductase inhibitors or statins is widespread in lipid-lowering therapies designed for AS. Statins reduce DNA damage via various mechanisms [154, 155, 156]. DNA impairment can facilitate apoptosis and result in untimely cell death [157, 158], both of which are primarily manifested in VSMCs in human AS. Thus, statins in AS have the potential to reduce VSMC apoptosis and senescence and maintain plaque stability. Mahmoudi *et al*. [159] demonstrated that atorvastatin accelerates DNA repair and attenuates impaired DNA function, cell aging, as well as telomere attrition within VSMCs via human double minute protein 2 (HDM2) phosphorylation, Nijmegen breakage syndrome protein (NBS-1) stabilization, and faster ataxia telangiectasia-mutated and histone H2AX phosphorylation, thereby promoting atherosclerotic plaque stability. Furthermore, pravastatin reportedly increases fibrous cap thickness and decreases VSMC apoptosis [52] (Table 1).

Baicalin and 6-shogaol affect VSMC apoptosis. The dry roots of *Scutellaria baicalensis* Georgi yield Baicalin, a flavonoid compound that has anti-proliferative functions involving different cell types. Baicalin promotes VSMC apoptosis by regulating maternally expressed 3 (MEG3)/p53 expression following treatment with ox-LDL [160]. Furthermore, the major biologically active compound 6-shogaol in ginger plays a role in curbing the oxidative stress-induced apoptosis of rat VSMCs. This inhibition is brought about by overexpressing the oxidation resistance 1 (OXR1)-p53 axis [53] (Table 1).

## 6. Autophagy of VSMCs

### 6.1 Autophagy of VSMCs During AS Development

Autophagy is an intracellular self-digestion and repair process that plays a significant role in protein degradation, exhaustion, and removal of damaged organelles, and is crucial for cell and tissue homeostasis. Recent research has shown that autophagy in VSMCs plays a significant role in defense, particularly the onset of AS [161], and acts as a pathway for cell death in AS. This process notably effects AS plaque instability in the late stage of the disease [162]. Disorders in VSMC autophagy are closely related to the development of AS.

### 6.2 Factors Affecting Autophagy of VSMCs and Their Mechanisms

**Fig. 6.**
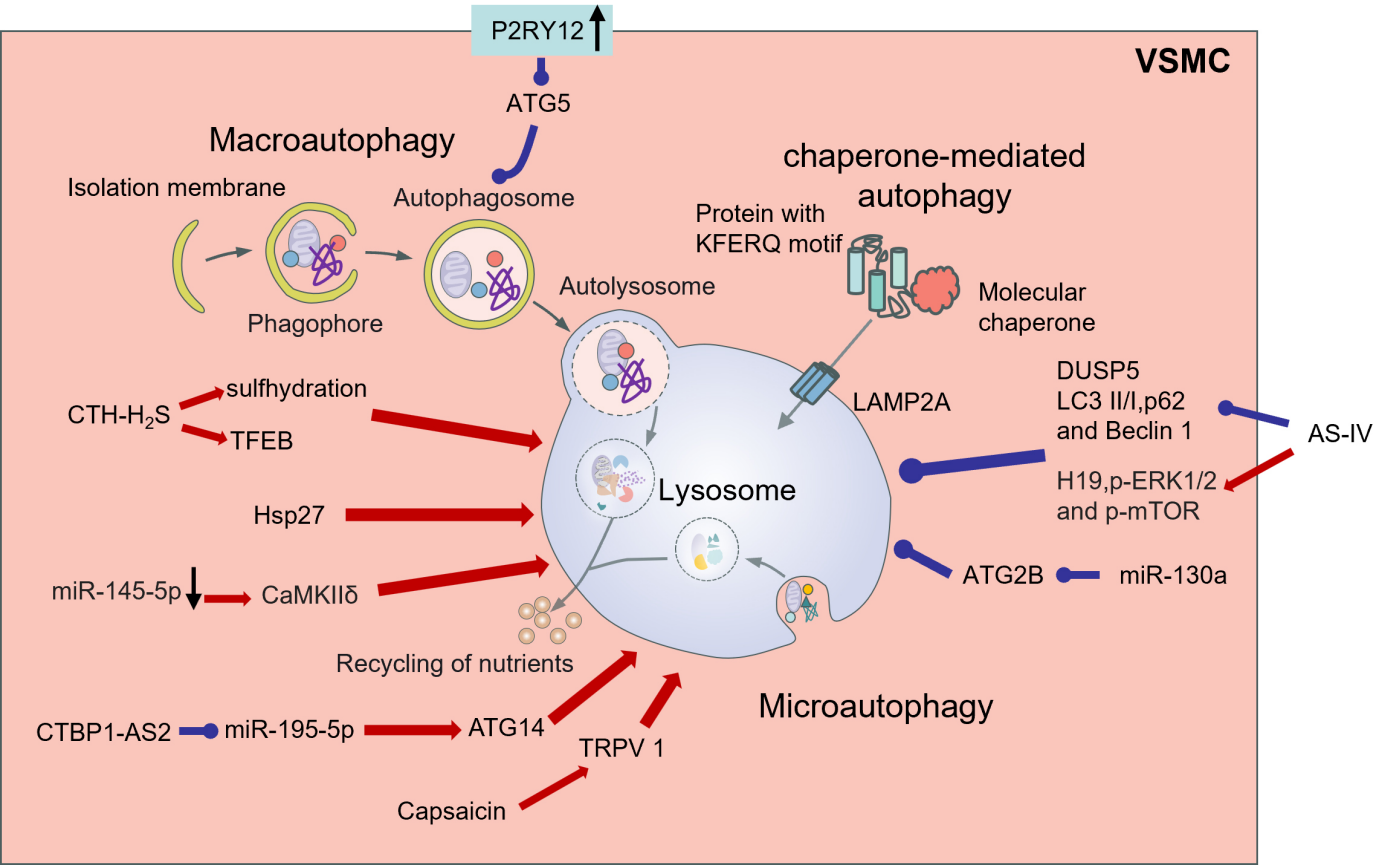
**Autophagy in VSMCs and its influencing factors**. VSMCs exhibit three autophagy modes: macroautophagy, microautophagy, and chaperone-mediated autophagy. In VSMCs, cystathionine gamma-lyase (CTH)-hydrogen sulfide (H_2_S) increases VSMC autophagy through sulfhydration, hydration, and activation of transcription factor EB (TFEB). The activation of P2RY12 downregulates the expression of ATG5, inhibiting the formation of autophagosomes in VSMCs. Small heat shock protein Hsp27 promotes autophagy to exert its anti-atherosclerosis effect. Astragaloside IV (AS-IV) inhibits DUSP5 and autophagy related proteins (LC3 II/I, p62 and Beclin 1), and increases the expression levels of H19, p-ERK1/2, and p-mTOR to inhibit autophagy of VSMCs in AS. miR-130a inhibits VSMC autophagy by inhibiting ATG2B. Downregulation of miR-145-5p promotes the expression of CAMkiδ and development of AS by activating autophagy in human VSMCs through the AMPK/mTOR/ULK1 signaling pathway. lncRNA CTBP1-AS2 promotes the expression of ATG14 and autophagy of VSMCs by inhibiting miR-195-5p. Capsaicin induces autophagy by activating transient receptor potential cation channel subfamily V member 1 (TRPV1). The red arrow represents promotion, the blue round head represents inhibition, the black arrow represents up-regulation or down-regulation, and the gray arrow represents the direction of the process. Created by Figdraw.

The fibrous caps formed by early VSMCs contribute to plaque stability, and VSMCs are a major source of cystathionine gamma-lyase (CTH)-hydrogen sulfide (H_2_S). CTH-H_2_S can reduce AS and plaque vulnerability by increasing VSMC autophagy through sulfhydration and activation of the transcription factor EB, thereby promoting collagen secretion and inhibiting apoptosis [163]. P2RY12 receptor regulates the autophagy pathway of VSMCs. The activation of P2RY12 receptor inhibits the formation of autophagosomes in VSMCs by down-regulating the expression of autophagy gene *ATG5*. The essential autophagy gene *ATG5* being knocked down results in a significant decrease in the cholesterol outflow of VSMCs induced by P2RY12 receptor inhibitors [30]. The small heat shock protein Hsp27 plays an inhibitory role in AS by modulating apoptosis and autophagy [164]. VSMC human antigen R protects against AS by increasing AMPK-mediated autophagy [165]. The autophagy defect of VSMCs is the main cause of AS. Autophagy defects in VSMCs lead to the upregulation of MMP9, TGFB, and CXCL12, thus accelerating aging and promoting post-injury neo-membrane formation and diet-induced AS formation [166]. Astragaloside IV (AS-IV) may inhibit DUSP5 and autophagy-associated proteins (LC3 II/I, p62, and Beclin 1); and H19 expression. p-ERK1/2 and p-mTOR levels are increased to inhibit autophagy and mineralization of VSMCs in AS [167]. Autophagy defects have in recent years been reported to promote the generation of plaques and result in unstable plaques due to VSMC-specific HuR knockdowns (Fig. 6) [165].

Numerous microRNAs are involved in the regulation of the pathological process of AS. miR-130a was found to inhibit autophagy in rat models, possibly inhibiting VSMC autophagy through ATG2B and thus inhibiting its proliferation. Therefore, miR-130a may be a potential therapeutic target for regulating autophagy in AS [168]. Downregulation of miR-145-5p promotes calcium/calmodulin-dependent protein kinase IIδ (CaMKIIδ) expression. Activation of autophagy in human VSMCs via the AMPK/mTOR/ULK1 signaling pathway promotes the development of AS [169]. lncRNA C-terminal binding protein 1 antisense RNA 2 (CTBP1-AS2) may regulate ATG14 through miR-195-5p to inhibit the proliferation of VSMCs and promote the autophagy of VSMCs, thus playing a role in AS (Fig. 6) [170].

Studies have suggested that autophagy is potentially involved in lipid metabolic processes [171, 172]. Autophagy activation decreases intracellular lipid accumulation; in contrast, autophagy suppression increases lipid accumulation in cells [171]. The death of VSMC and VSMC-derived foam cells impairs plaque stability, in contrast, autophagy facilitates cell survival. VSMC autophagy in *ApoE*^-⁣/-^ mice stimulates cholesterol outflow and inhibits lipid accumulation and necrotic core formation [173]. Therefore, autophagy plays a potential role in regulating VSMC-derived foam cell formation. For example, capsaicin stimulates the function of transient receptor potential cation channel subfamily V member 1 (TRPV 1) to promote autophagy and restrain the formation of foam cells originating from VSMCs via adenosine monophosphate (AMP)-activated protein kinase signaling [174]. In summary, autophagy is a protective mechanism against the aging and death of VSMCs; However, the synthesis of excessive activation of autophagy may be triggered by VSMC phenotype and aggravate AS [175] (Fig. 6).

### 6.3 Modulators Affecting Autophagy of VSMCs

Paeonol: Paeonol is isolated from the basal bone of the Mutan cortex and has anti-atherosclerosis and anti-apoptosis effects. Paeonol can induce autophagy of VSMCs by activating the Class III PI3K/Beclin-1 signaling pathway, and ultimately inhibit the apoptosis of VSMCs [54] (Table 1).

Celastrol: In the field of traditional Chinese medicine research, Celastrol, which is a quinine methyl ether triterpenoid compound sourced from the root bark of tripterygium wilfordii, has extensive biological properties, which include anti-obesity, the protection of cardiovascular health, anti-inflammatory actions, and antioxidant activities. Celastrol plays an antioxidant role in various cell types and enhances autophagy, which regulates reactive oxygen species (ROS) that promote cell aging. A previous study suggested that celastrol may reduce ROS production by activating autophagy to counteract VSMC senescence and successfully prevent and treat AS [55] (Table 1). In addition, celastrol upregulates the expression of ABCA1 by activating LXRα and inhibits lipid deposition in VSMCs. At the same time, celastrol decreases lipid accumulation by activating autophagy in VSMCs [56] (Table 1).

Nicotine: Smoking is an established risk factor for AS. Nicotine, as the main component of cigarettes, induces autophagy, which in turn promotes the transformation of VSMC phenotypes and accelerates the atherosclerotic process [176]. In addition, elevated cathepsin S (CTSS) plays a key role in the progression of AS. Nicotine activates autophagy in VSMCs and AS plaques, inhibits mammalian rapamycin Complex 1 target (mTORC1) activity, promotes nuclear translocation of the transcription factor EB (TFEB), and promotes the synthesis and secretion of CTSS. Therefore, CTSS inhibitors can inhibit nicotine-induced AS *in vivo*. Nicotine-regulated VSMC autophagy disturbances were observed to induce and enhance to formation of AS [177].

## 7. Conclusion

The role of VSMCs in AS has stimulated an increasing number of researchers to investigate the pathological changes of VSMCs in the progression of AS, along with exploring various targets, signaling pathways, and agents that affect their function. Numerous studies indicate that foam cells derived from VSMCs play a crucial role in the progression of AS, particularly in advanced plaques where most lipid accumulation originates from VSMCs. Additionally, the phenotypic transition, proliferation, migration, apoptosis, and autophagy of VSMCs play diverse roles in the formation, repair, and rupture of AS plaques. Therefore, maintaining healthy VSMC function is essential to protect vessel walls from AS. The factors and mechanisms of action that regulate VSMCs in AS are diverse, and further research is necessary to develop new therapies to prevent and limit the progression of AS. As the understanding of the roles of VSMCs in AS continues to expand, there is an opportunity to develop novel therapeutic approaches for the clinical treatment of AS, thereby reducing the incidence of life-threatening major acute events that are associated with AS.

## 8. Future Perspective

### 8.1 Emerging Therapeutic Strategies Targeting VSMCs in AS

Compared to traditional treatments such as lipid-lowering, blood pressure control, antiplatelet, and anticoagulant therapies, research focusing on VSMC targets and modulators provides a more thorough understanding of the pathological processes of AS. Previous treatment strategies mainly targeted foam cells derived from macrophages, without considering VSMC-derived foam cells. However, targeting VSMC-derived foam cells complements and updates existing AS treatment methods, offering more viable options for AS therapy.

Despite increasing research and knowledge on VSMCs in AS, macrophages and VSMCs play significant roles in the process of AS development. Studies showing that VSMC-derived foam cells may even outnumber those derived from macrophages in advanced AS stages. Extensive research on VSMCs has identified various changes, including phenotypic switching, proliferation, migration, apoptosis, and autophagy, which impact the formation and progression of atherosclerotic plaques. Maintaining a healthy contractile phenotype in VSMCs can prevent and inhibit the occurrence and progression of AS. Conversely, VSMC apoptosis affects fibrous cap thickness and alterations in plaque composition, thus influencing the stability of advanced AS plaques. VSMC proliferation, migration, and autophagy play dual roles in AS progression. On one hand, VSMC proliferation and migration promote AS development while contributing to fibrous cap formation to prevent plaque rupture. On the other hand, VSMC autophagy serves a defensive role in maintaining cellular homeostasis during early AS stages; however, as a form of cell death, autophagy affects fibrous cap thickness and plaque stability in advanced AS stages.

Researchers have conducted numerous *in vivo* and *in vitro* experiments in response to these findings, identifying numerous targets and modulators that can be used to treat AS. They have discovered numerous regulators that affect VSMC function, providing new areas for treatment for AS patients. Compared to traditional treatments such as lipid-lowering, blood pressure reduction, antiplatelet therapy, and anticoagulation, the study of these targets and modulators that target VSMCs provides a more thorough and detailed understanding of the pathological processes of AS. VSMC-derived foam cells are indispensable in the advancement of AS plaques, even dominating the foam cells in advanced stages. Targeting foam cells in plaques unquestionably inhibits the development of AS to a certain extent. Researchers have thoroughly studied foam cells derived from macrophages and identified many potential targets. Targeting foam cells derived from VSMCs with specific targets and modulators can supplement and update existing AS treatments. Known for their crucial role in gene expression regulation, microRNAs participate in diverse biological procedures, such as cell proliferation, apoptosis, differentiation, metabolism, along with immune response. Therefore, they play a crucial role in the occurrence and development of numerous diseases, particularly vascular diseases. Consequently, microRNAs are key regulatory factors in AS and are prominently featured in the discussion of VSMC phenotypic switching, proliferation, migration, and autophagy. In particular, recent research on VSMC phenotypic switching and proliferation migration has predominantly focused on microRNAs, highlighting their significance in current research. These findings contribute to the identification of new treatment strategies for AS and for more efficacious therapies.

### 8.2 Challenges and Future Research Directions in Treating AS Targeting VSMCs

An increasing number of researchers have recognized VSMCs as crucial cells in exploring therapeutic approaches for AS. With the increasing investment in research and technological advancements, the focus on VSMCs in AS is expanding. However, most therapeutic strategies targeting VSMCs are still in the early stages of basic animal and cell experiments, lacking sufficient clinical data to support their effectiveness and safety in the treatment of AS. Biological differences between humans and animals prevent direct application of various research results to humans, necessitating further investment and research to optimize treatment strategies for clinical practice. Nevertheless, animal models still hold significance in describing important pathways and fundamental principles that may lead to the development of human atheroma. Furthermore, the function of VSMCs in AS is complex, involving various aspects such as plaque formation, stability, and rupture. Therefore, a single therapeutic target may not fully cover all the functions of VSMCs, thus necessitating the adoption of a multi-targeted therapy strategy to enhance treatment efficacy. Additionally, intricate interactions are observed between VSMCs and other cell types (such as inflammatory cells, endothelial cells), which may influence the function and phenotypic transformation of VSMCs. Consequently, these intercellular interactions need to be considered in developing therapeutic strategies targeting VSMCs and may require the comprehensive investigation of multiple cell types as therapeutic targets. Moreover, the heterogeneity of VSMCs may lead to uncertainty in treatment outcomes. VSMCs may exhibit different phenotypes in different arterial locations and pathological states. Therefore, this heterogeneity needs to be considered in therapeutic strategies targeting VSMCs and may necessitate different treatment approaches towards different types of VSMCs.

Future research should focus on addressing these challenges. First, clinical studies need to be reinforced to validate the effectiveness and safety of therapeutic strategies targeting VSMCs and promote their clinical application. Second, exploring multi-targeted therapy strategies to improve treatment efficacy can be a promising approach. Third, investigating the impact of intercellular interactions on VSMC function can help optimize treatment protocols. Further elucidating the mechanisms underlying VSMC functionality and phenotypic switching can lead to the identification of novel therapeutic targets. Furthermore, following the rapid advancement of scientific technology, our understanding of the development and progression of human AS disease has improved. High-throughput sequencing analysis and genetic studies show potential in the identification of optimal treatment targets and methods tailored to AS patients.

## Abbreviations

ATF4, activating transcription factor 4; ACAT1, acyl Coenzyme A cholesterol acyltransferase 1; AMP, adenosine monophosphate; ART, artemisinin; AS, atherosclerosis; ABC, ATP-binding cassette; CHOP, C/EBP homologous protein; CARMN, cardiac mesoderm enhancer-associated non-coding RNA; CBX3, chromobox protein homolog 3; CircMAPK, circular mitogen-activated protein kinase; CircMTO, circular RNA mitochondrial translation optimization; SR-A, class A scavenger receptor; CD, cluster of differentiation; COX2, cyclooxygenase-2; DOT1L, disruptor of telomeric silencing 1-like; ETS, erythroblast transformation specific; LKO-Erα, estrogen acts on hepatocyte estrogen receptor alpha; FOXO, forkhead box O; GLP1, glucagon-like peptide-1; GSK-3, glycogen synthase kinase-3; GAS, growth arrest-specific; G6PD, glucose-6-phosphate dehydrogenase; HDAC9, histone deacetylase 9; HDM2, human double minute protein 2; H_2_S, hydrogen sulfide; IGF1, insulin-like growth factor 1; IQGAP 1, IQ motif-containing GTPase activating protein 1; KLF4, Kruppel-like factor 4; LXRα, liver X receptor alpha; lncRNAs, long non-coding RNAs; mTORC, mammalian target of rapamycin complex; MEG3, maternally expressed 3; miRNAs, microRNAs; MEF2C, myocyte enhancer factor 2C; NADPH, nicotinamide adenine dinucleotide phosphate; NBS-1, Nijmegen breakage syndrome protein; NF-κB, nuclear transcription factor kappa B; Oct4, octamer-binding transcription factor 4; OXR1, oxidation resistance 1; ox-LDL, oxidized low-density lipoprotein; PPARδ, peroxisome proliferator-activated receptor delta; PI 3-kinase, phosphatidylinositol 3-OH kinase; IP receptor, prostacyclin receptor; PGD2, prostaglandin D2; PGE2, prostaglandin E2; PGF2α, prostaglandin F2 alpha; PGI2, prostaglandin I2; RUNX2, runt-related transcription factor 2; SRF, serum response factor; SNHG12, small nucleolar RNA host gene 12; SPRR3, small proline-rich repeat protein; MYH11, Myosin Heavy Chain 11; NaHS, sodium thiosulfate; SAMd 1, sterile alpha motif domain 1; TXA2, thromboxane A2; TLR4, toll-like receptor 4; TNFα, tumor necrosis factor alpha; VSMCs, vascular smooth muscle cells; LDLR, low-density lipoprotein receptor; LRP1, low-density lipoprotein receptor-related protein 1; ag-LDL, aggregated LDL; FLN-A, filamin-A; LOX-1, lectin-like oxidized low-density lipoprotein receptor-1; TGF-β, transforming growth factor-β; BMSCs, bone marrow mesenchymal stem cells; TREM2, triggering receptor expressed on myeloid cells 2; LKB1, liver kinase B1; HDGF, hepatoma-derived growth factor; TM6SF2, transmembrane 6 superfamily 2; CTH-HS, cystathionine gamma-lyase-hydrogen sulfide; TFEB, transcription factor EB; P2RY12, P2Y12 receptor; Hsp27, heat shock protein 27; TGFB, transforming Growth Factor Beta; CXCL12, C-X-C motif chemokine ligand 12; AS-IV, astragaloside IV; DUSP5, dual specificity phosphatase 5; CaMKIIδ, Calcium/calmodulin dependent protein kinase IIδ; CTBP1-AS2, C-terminal binding protein 1 antisense RNA 2; TRPV 1, transient receptor potential cation channel subfamily V member 1; ROS, reactive oxygen species; CTSS, cathepsin S.
